# Suppression of *Escherichia coli* O157:H7 by Dung Beetles (Coleoptera: Scarabaeidae) Using the Lowbush Blueberry Agroecosystem as a Model System

**DOI:** 10.1371/journal.pone.0120904

**Published:** 2015-04-07

**Authors:** Matthew S. Jones, Shravani Tadepalli, David F. Bridges, Vivian C. H. Wu, Frank Drummond

**Affiliations:** 1 University of Maine, School of Biology and Ecology, Orono, ME, United States of America; 2 Washington State University, Department of Entomology, Pullman, WA, United States of America; 3 University of Maine, School of Food and Agriculture, Orono, ME, United States of America; 4 University of Maine, Cooperative Extension, Orono, ME, United States of America; USDA-ARS-ERRC, UNITED STATES

## Abstract

Wildlife as a source of microbial contamination is a food safety concern. Deer feces (scat) have been determined as a point source for *Escherichia coli* O157:H7 contamination of fresh produce. The ecological role of the scooped scarab (*Onthophagus hecate* (Panzer)), a generalist dung beetle species common in Maine blueberry fields, was explored as a biological control agent and alternatively as a pathogen vector between deer scat and food.

A large-scale field survey of wildlife scat indicated that pathogenic *E*. *coli* O157:H7 was present, albeit at a low prevalence (1.9% of samples, n = 318), in the Maine lowbush blueberry agroecosystem. A manipulative field experiment verified that, should contact occur between deer scat and blueberry plants and fruit during the summer, contamination with *E*. *coli* O157:H7 can occur and persist for more than 72 h. For both the positive control and an experimental scat inoculation treatment, the levels of the bacterial population decreased over time, but at different rates (treatment x time interaction: *F*_(1.9,18.8)_ = 358.486, *P* < 0.0001). The positive control inoculation, which resulted in a higher initial *E*. *coli* level on fruit, decayed at a faster rate than inoculation of fruit via scat in the experimental treatment.

We conducted 2 laboratory studies to elucidate aspects of dung beetle feeding ecology as it relates to suppression of *E*. *coli* O157:H7 from deer scat to lowbush blueberry fruit. In both experiments, dung beetles buried the same amount of scat whether or not the scat was inoculated with the pathogen (*F*_(1,6)_ = 0.001; *P* = 0.999 and (*F*_(2,17)_ = 4.10, *P* = 0.147). Beetles feeding on *E*. *coli* inoculated deer scat were not found to vector the pathogen to fruit. In two studies, beetles lowered the amount of pathogenic *E*. *coli* persisting in soils compared to soils without beetles (*F*_(2,9)_ = 7.757; *P* = 0.05 and *F*_(2,17)_ = 8.0621, *P* = 0.004).

Our study suggests that the dung beetle species, *Onthophagus hecate*, has the potential to contribute to the suppression of *E*. *coli* O157:H7 in agricultural landscapes.

## Introduction

Transmission of *Escherichia coli* O157:H7 to humans may occur through consumption of contaminated raw foods or by direct or indirect exposure to infected fecal material [1]. Many wildlife species are reservoirs of pathogens that threaten domestic animal and human health; therefore, understanding the behavior and biological interactions between wildlife and food-production units is critical [2]. Wildlife may be reservoirs for *E*. *coli* O157:H7 in the agricultural environment, or they may be vectors involved in the contamination of plants directly by scat (feces deposition) and indirectly by scat contamination of surface waterways or soil [3]. The role of wildlife as a source of foodborne microbial contamination along the farm-to-fork continuum is a longstanding concern among public health and food safety agencies [4]. During production, there are many opportunities for food contamination to occur via irrigation, soil, equipment, fieldworkers, and food handlers [5]. Despite these possible sources of contamination, it is hypothesized that the prevalence of this pathogen is underestimated in the U.S. food supply [6].

*Escherichia coli* O157:H7 outbreaks attributed to contamination of fresh produce in agricultural fields with white-tailed deer scat have occurred on multiple occasions [7]. Additionally, harboring of *E*. *coli* O157:H7 in white-tailed deer (*Odocoileus virginianus* Zimmermann) populations has been studied, though mostly outside of the context of agriculture. Deer scat has been identified as a possible source of contamination in outbreaks of *E*. *coli* O157:H7 stemming from both unpasteurized apple juice and apple cider [8,9]. Most recently, deer scat was determined to be the source of contamination of strawberries in Oregon [10]. The known presence of O157:H7 in deer scat has implications for developing management programs to control this pathogen on the farm [11]. From a food safety standpoint, it is important to understand the implications of wildlife in and around fresh produce crop fields.

The ability of dung beetles to suppress pests and pathogens in pasture ecosystems has long been recognized [12–14]. Nichols et al. describe the multiple benefits dung beetles can provide, including suppression of enteric parasites, decreases in parasite dispersal, as well as reduction of dung-dwelling flies [15]. Ryan et al. found that widespread dung burial by *Bubas bison* (Coleoptera: Scarabaeidae) could substantially reduce pathogens (*Cryptosporidium* oocytes) and that *B*. *bison* may be an ideal candidate as a biological control agent [16]. However, Xu et al. [17] found *E*. *coli* O157:H7 on the exterior of a dung beetle (Coleoptera: Scarabaeidae) that had previously fed on pathogen-laden scat, thus implying dung beetles could act as a pathogen vector.

Data suggest that environmental variables associated with blueberry fields drive insect-mediated deer scat degradation; complete removal of deer scat, placed experimentally within Maine blueberry fields, has been observed in periods less than 7 days [18]. To more clearly understand and apply this information, a better understanding of the role of dung beetles is needed as a potential biological suppressor of the pathogen, as a potential vector for the pathogen between scat and fruit, and as a potential reservoir for the pathogen. We chose to use the lowbush blueberry agroecosystem to test multiple, broadly applicable questions concerning the natural suppression/transmission of pathogenic *E*. *coli* O157:H7 from white-tailed deer scat to lowbush blueberry fruit. This system was chosen for the following reasons: Lowbush blueberry is a low perennial plant (35 cm) that forms dense mats covering entire fields. These fields are most often embedded within a dense forest matrix, encouraging both the foraging and the movement of wildlife in and around fields and increasing the risk of food contamination. White-tailed deer scat are commonly seen within and around blueberry fields [18,19]. Given the short structure of these blueberry plants and likely contact with white-tailed deer scat, contamination of berries is plausible. No one has explored how dung beetle-mediated deer scat removal might influence the risk of fruit contamination from pathogen-laden scat. To our knowledge, there have not yet been any cases of pathogenic *E*. *coli* related illness in the lowbush blueberry agroecosystem.

Time-lapse, macro-video imaging of dung beetle feeding in our study area, in addition to linear pitfall trapping [18,20], indicate that the scooped scarab, *O*. *hecate* Panzer (Coleoptera: Scarabaeidae), is the dung beetle species most biologically relevant for this study. *Onthophagus hecate* is a generalist found feeding on a diversity of wildlife, livestock, and human scat, as well as rotting fungi, fruit, and carrion. Additionally, *O*. *hecate* has the largest geographical distribution of any dung beetle in North America [21].

To better understand the risk of scat contamination of produce and the ecological role of dung beetles in food safety, the objectives of this project were to 1) investigate the prevalence of pathogenic *E*. *coli* O157:H7 in wildlife (scat) present within the Maine lowbush blueberry agroecosystem, 2) understand if transmission of *E*. *coli* results from direct contact between contaminated deer scat and fruit and if so, how long the contamination persists on fruit, 3) study the difference of dung beetle feeding preferences for white-tailed deer scat inoculated with *E*. c*oli* O157:H7 compared to non-contaminated deer scat, and 4) understand the role of dung beetles in suppressing/vectoring *E*. *coli* O157:H7 from white-tailed deer scat to berries and soil, as well as the persistence of *E*. *coli* O157:H7 in the soil.

## Materials and Methods

### Survey of wildlife scat in lowbush blueberry

Twelve blueberry farms throughout the major production regions in Maine (Waldo, Hancock, and Washington counties) were surveyed, with landowner permission, for wildlife scat throughout the growing season. Surveys took place in each field, three times, representative of the spring, summer, and fall seasons. Sampling was conducted in April, June, and August/September (2012). Each field’s perimeter was examined for wildlife scat. White-tailed deer, black bear, snowshoe hare, and turkey scat were most commonly detected. Field interiors were also examined during the April sampling period (before plants leafed out) by walking “W-shaped” transects [22]. Scat samples with a ruler were photographed to assist in identification. Each scat sample was collected with a new set of sterile gloves and placed in a sterile, 18 oz. roll-top bag (FisherBrand, Pittsburg, PA) and immediately transferred, on ice, from field site to refrigerated storage (4°C) at the University of Maine. In the lab, each of these original scat samples (n = 318) were diluted (1:5) in Phosphate Buffer Saline (PBS) based on their mass and homogenized for 2 min using a stomacher (Tekmar Company, Cincinnati, OH). One ml of these individual samples were pooled together into groups (based on field site of origin and collection date) yielding 36 pooled samples for analysis. Initial screening for *E*. *coli* O157:H7 was carried out on these 36 pooled scat samples. Subsequently, if a pooled sample tested positive for *E*. *coli* O157:H7, each individual sample comprising the larger pooled sample was screened individually for the presence of *E*. *coli* O157:H7. Isolation and identification of *E*. *coli* O157:H7 was carried out following the FDA/BAM manual with some modifications [23]. Briefly, samples were enriched in modified trypticase soy broth with novobiocin (mTSB) at 37°C for 24 h and were later isolated on sorbitol MacConkey agar (SMAC) supplemented with potassium tellurite and cefixime (CT)(Dynal, Lake success, N.Y.). Samples presumed to be *E*. *coli* O157:H7 positive were tested by biochemical characterization and finally confirmed for O157:H7 antigen by Remel RIM O157 and H7 serological latex agglutination test (Remel, Lenexa, KS). *E*scherichia *coli* O157:H7 ATCC 35150 was used as a positive control.

### Direct transmission of pathogen field study design

In order to test the potential for infected deer to directly transmit *E*. *coli* O157:H7 to lowbush blueberries, a field study was designed using an attenuated, two-strain inoculum of *E*. *coli* O157:H7 “cocktail” (ATCC 700728 and B6914) as a surrogate for the pathogenic strains of this serotype [24,25]. Preliminary lab studies indicate that this cocktail persisted in the environment at the same levels as pathogenic *E*. *coli* O157:H7 (ATCC 35150), verifying its effectiveness as an attenuated strain for the field study. In addition, Kudva et al. [26] found that lack of shiga toxin type 1 and 2 genes in *E*. *coli* O157:H7 had little or no influence on bacterial survival under field conditions. This field study took place from July 15–18, 2013, at Blueberry Hill Farm (44.647008, −67.649825), a research farm managed by the University of Maine. An experimental field plot consisted of twenty-four 1m x 1m subplots of lowbush blueberry, separated from one another by 1m strips. Within each subplot, the area with the highest density of berries was flagged. Before applying treatments to the berries, a bottomless 26 cm diameter plastic bucket was lowered around plants to be treated in order to prevent bacteria drift/cross contamination. All subplots possessed ripe blueberries at the time of the experiment. Three treatments were applied to randomly selected experimental subplots. First, a positive control containing attenuated *E*. *coli* cocktail in aqueous solution (final inoculum: approximately 6 log CFU/g) was sprayed onto berries and leaves using a standard spray bottle (Zep Inc. Marietta GA) to ensure bacterial contact with berries, to confirm laboratory methods, and to measure persistence of the pathogen in the environment. Second, an experimental treatment contained white-tailed deer scat inoculated with the attenuated *E*. *coli* O157:H7 (final inoculum: approximately 6 log CFU/g with equal amounts of both inoculum strains). The experimental treatment scat pellets were inoculated using a dipping method. Around 70 pellets for each plot were dipped in 70ml of the attenuated *E*. *coli* O157:H7 strain cocktail and were shaken for 3 min using a shaker at 120 rpm (Barnstead Thermolyne, Roto Mix-Type 50800). After 3min of shaking, the inoculum was drained from the scat and they were dried for 2 h. Fresh white-tailed deer scat was collected from the University of Maine Experimental Forests (Orono, ME). Third, a negative control contained non-inoculated, sterile water sprayed directly onto plants in place of attenuated *E*. *coli*.

For the experimental group, 28 g (around 70 pellets) of fresh white-tailed deer scat inoculated with attenuated *E*. *coli* O157:H7 was dropped directly onto blueberry plants (leaves and berries) from approximately 1 meter above the plant canopy within randomly assigned subplots, taking care to initiate as much contact between scat and berries as possible. Twenty-eight g was the approximate weight of 68.7 scat pellets, the number of pellets per pellet-group in an average white-tailed deer scat sample [27]. At the time of deployment, the wind speed was 3.2 km/h and the temperature was 17°C.

Two hours after the treatments were applied to the wild blueberry plants (control treatments had dried), roughly 15 g of treated berries were collected from each subplot (n = 24). Blueberries were harvested by clipping stems, avoiding contact with the berries, and were placed in sterile, 18 oz. roll-top bags (FisherBrand). Berries were immediately transferred to a cooler with ice packs for transport to the lab. Forceps and scissors were sterilized using 95% ethyl alcohol (Pharmco products INC, Brookfield CT) to ensure no cross-contamination occurred between replicates. This collection process was repeated 24, 48, and 72 h after inoculation and bacterial enumeration was conducted within 6 h of sample collection.

Sample processing involved aseptically separating 15 g of blueberries from stems, adding15ml of 0.1% peptone water, and shaking for 3 min using a shaker at 120 rpm (Barnstead Thermolyne, Roto Mix-Type 50800). Serial dilutions of this solution were plated on Cefixime Potassium Tellurite Sorbitol-MacConkey Agar (CT-SMAC, Neogen, Lansing, MI, USA). Plates were incubated at 37°C overnight, and then *E*. *coli* counts were enumerated. At least two presumptive colonies were screened for the presence of O157 and H7 antigen by latex agglutination (Remel RIM,Lenexa, KS). A MANOVA/ANOVA, with time as the repeated measure (dependent variables) and treatment as the categorical independent variable, was used to analyze the differences in logarithm transformed numbers of colony-forming units (CFU) on the berries harvested from each treatment. Mauchly’s sphericity test was used to determine if the conservative Greenhouse-Geisser correction was needed to adjust degrees of freedom [28].

### Role of the dung beetle in pathogen transmission/suppression

To better understand the role of the dung beetle, *Onthophagus hecate*, in the potential suppression/transmission of *E*. *coli* O157:H7 from contaminated scat to blueberries and soil, two laboratory experiments were conducted with three treatment combinations in microcosms. Treatment one included 4 beetles and 6 deer pellets (scat) inoculated with *E*. *coli* O157:H7. Treatment two included 4 beetles and 6 deer pellets without *E*. *coli* O157:H7. Treatment three included 6 deer pellets inoculated with *E*. *coli* O157:H7 without beetles.

The first lab experiment was designed to provide a laboratory simulation of field conditions, and took place took place in 37.9-liter glass microcosms (Great Choice, PetSmart, Phoenix, AZ) filled with fruit-bearing lowbush blueberry plants including intact soil from blueberry fields. The second lab experiment used 600 ml beakers with autoclaved soil, instead of blueberry plants/soil, and this provided a more direct examination of the beetle/pathogen interaction.

#### Collection of blueberry plants from field (for lab experiment 1)

Twelve sections of mature, fruit-bearing, lowbush blueberry “sod” (25 cm wide x 50cm long x approximately 10 cm deep) were carefully cut from fields at Blueberry Hill University Research Farm (Jonesboro, ME). Plant and soil structure were kept intact, as this has been shown to influence dung beetle competitive interactions and feeding [29]. Blueberry plants were fit into twelve sterilized glass terrariums.

#### Collection of soil (for lab experiment 2)

Soil was collected from a blueberry field at Blueberry Hill Farm in Jonesboro, ME. Rocks and roots were removed. Soil (300ml) was placed in 600 ml glass beakers and autoclaved. Aluminum foil covered the microcosms for the duration of the experiment.

#### Collection and storage of beetles (for both lab experiments)

For experiments 1 and 2 conducted in mid-July 2012 to mid-June 2013, dung beetles (*O*. *hecate*) were collected using live pitfall traps consisting of 11 cm (diam.) x 7.5 cm (depth) deli cups buried flush with the soil surface and filled with 2 cm of dried deer scat. A mesh bag containing human dung was suspended directly above each cup (modified from [20]). Traps were placed within blueberry fields (Frankfort and Stockton Springs, ME) as well as in a pasture containing horses and cattle located on the University of Maine Campus (Orono, ME). All beetles collected for experiments were verified as *Onthophagus hecate* [30]. Beetles collected were maintained at 25°C and were regularly fed fresh white-tailed deer scat. All *O*. *hecate* were reared for four days together. Twenty-four hours prior to the initiation of the experiment, food was taken from dung beetles, which were then sorted by sex (major male form, minor male form, female). Groups composed of 2 females, and 2 males (1 major male, possessing a large bifurcated horn on the pronotum, and 1 minor male, lacking horn) were held together overnight before being added to experimental enclosures.

#### Collection of deer scat (for both lab experiments)

Fresh white-tailed deer scat pellets were collected from the University Experimental Forest (Orono, ME) within 2 months prior to each experiment. Scat was stored in a freezer at -17°C.

#### Surface-sterilization of beetles (for lab experiment 2)

In order to minimize the amount of microflora introduced to experimental microcosms, dung beetles were surface-sterilized using household bleach with 6% sodium hypochlorite (Great Value Brand, Walmart, Bangor, ME) (500 rpm, for 30 seconds) which did not influence mortality or behavior of the beetles according to preliminary studies. Beetles were surface-sterilized within one-hour prior to use in experimentation.

#### Bacteria Inoculum Preparation (for both lab experiments)

No reports on specific concentration of the fecal shedding load of *E*. *coli* O157:H7 was found for white-tailed deer, so a concentration range found in sheep scat naturally laden with *E*. *coli* O157:H7 was used as a proxy/baseline. The scat from the two animals is very similar in composition and shape. Research performed by Kudva et al. [26] examined the fecal shedding of *E*. *coli* O157:H7 in sheep and found a range of <10^2^ to 10^6^ CFU/g. To simulate the scat of an infected white-tailed deer, fresh deer scat pellets were intentionally inoculated with an *E*. *coli* O157:H7 cocktail of similar concentration (10^7^ CFU/g). Two different strains of pathogenic *E*. *coli* O157: H7 (ATCC 35150 and ATCC 700594) were transferred from stock culture stored at -80°C into 10ml BHI broth, and allowed to grow overnight before they were centrifuged at 15,300 × *g* for 15 min at 4°C and then washed twice with 10 ml of sterile buffered peptone water and re-suspended in 10 ml of Buffer Peptone Water (Bacto, Becton Dickinson, Sparks,MD, USA). The two *E*. *coli* O157: H7 strains were combined with equal volumes to yield 20 ml of a cocktail mixture.

#### Inoculation of Scat Samples

White-tailed deer scat pellets were spot inoculated with 200μl of previously described pathogenic *E*. *coli* O157:H7 cocktail. The initial level of inoculum on scat pellets was 7 log CFU/g after completion of spot-inoculation. A control group of scat pellets, which were inoculated with sterile water, was also included in the study. To check the viable cell count of these scat samples, 6 bacteria-cocktail-inoculated scat pellets were placed into a sterile stomacher bag with 10 ml of 0.1% peptone water (Bacto, Becton Dickinson, Sparks,MD, USA) and stomached for 2 min. Serial dilutions from these 6 scat samples were then plated on Macconkey Sorbitol Agar (MSA, Neogen).

Treatment groups were set up in a randomized block design with 4 replications. Blocks were arranged perpendicular to a window in the lab, which could potentially be viewed as a stratification variable. Appropriate elements (inoculated scat pellets, control scat pellets, and beetles) for each treatment were delivered to respective enclosures. Scat was placed directly on the soil between blueberry plants. If a treatment included dung beetles, these were delivered adjacent to scat. Enclosures were covered securely with fine window screening to provide adequate ventilation to plants. Beetles were allowed the opportunity to feed on feces for 10 days (21°C, 24 h light) and monitored daily. After beetles were allowed to feed, the amount of feeding by beetles was calculated from the percentage of deer pellets remaining on the soil surface.

For experiment 1, all ripe/harvestable blueberries were harvested from each enclosure, from which 25 g were randomly selected for analysis. Within each enclosure, 2 cm (width) x 7.5 cm (depth), soil cores were taken immediately adjacent to where scat had been placed until 25 g of soil were obtained. All soil and blueberry samples were then tested for the presence of *E*. *coli* O157:H7 by direct selective plating on MSA [23]. Uneaten scat, remaining on the soil surface within inoculated treatment group tanks, was also tested for the presence of *E*. *coli* O157:H7 on day 10, post-experiment initiation.

For experiment 2, the entire amount of soil (300 ml) in the beaker, as well as all scat, were analyzed for levels of pathogenic *E*. *coli* O157:H7. Beetles were also collected from microcosms for analysis. Since soil for experiment 2 was sterilized before the experiment, any pathogens found in the microcosm system are the result of transmission/incorporation from scat.

#### Testing for E. coli O157:H7 in blueberries, scat, and soil

Each 25 g of soil sample and 25 g blueberry sample from each group were transferred into individual sterile stomacher bags. To each stomacher bag 25 ml of 0.1% peptone water was added (Bacto, Becton Dickinson, Sparks, MD, USA) and homogenized for 2 min using a stomacher (Tekmar Company, Cincinnati, OH). Homogenates were serially diluted in sterile 0.1% peptone water, and spread-plated in duplicate on SMAC without supplements (Neogen, Corporation, Acumedia, Lansing, MI) for blueberries and soil and on Tryptic Soy Agar (TSA, Trypticase Soy Agar, Corporation, Acumedia, Lansing, MI) for soil only and incubated at 37°C for 24h. At least two presumptive colonies on SMAC were screened for the presence of O157 and H7 antigen by latex agglutination (Remel RIM,Lenexa, KS)[23].

#### Scat removal analysis

The percentage of scat removal after 10 days of feeding was calculated across all treatments by recording the amount of the deer pellets that had been consumed/buried in each of the tanks. A one-way ANOVA was used to compare the percentage removal of scat pellets across Beetle with *E*. *coli* O157:H7 and Beetle without *E*. *coli* O157:H7 treatments. Analysis was performed on untransformed data, as raw data were homoscedastic and normally distributed.

#### Blueberry contamination analysis (lab experiment 1 only)

No statistical analysis could be attempted, as *E*. *coli* was never detected in any of the treatments.

#### Soil contamination analysis

A one-way ANOVA was used to compare the amount of colorless bacterial colony forming units (measured in CFU’s) persisting in the soil samples from all treatment group tanks. For this analysis, data (CFU’s) were rank-order transformed in order to meet the assumptions of being normally distributed and having homogeneity of variance. *E*. *coli* O157:H7 contamination was determined as presence/absence.

## Results

### Survey of wildlife scat in lowbush blueberry

Two pooled samples (of 36 total) were determined to be *E*. *coli* O157: H7 positive. Samples composing these pooled samples were collected from a Washington Co., ME field site in June and a Waldo Co., ME field site in September. One of the four individual scat samples was positive for *E*. *coli* O157:H7 from the positive pooled Washington Co. sample. This sample was of white-tailed deer origin. Five of the 20 individual scat samples were positive for *E*. *coli* O157:H7 from the positive pooled sample from Waldo Co. Positive samples were identified as white-tailed deer (2), wild turkey (1), and unknown (2).

### Direct transmission of pathogen field study

**Fig 1:**
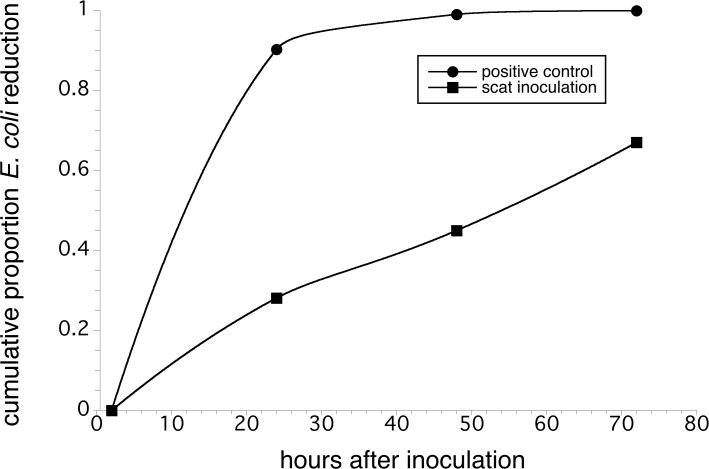
Field Experiment- Reduction in *E*. *coli* levels over time. Decay of *E*. *coli* O157:H7 mixture (strains ATCC 700728 and B6914) in field inoculation study. Reductions in *E*. *coli* levels on fruit are proportions of the inoculum at the 2 hr sampling period (positive control log CFU / g fruit = 6.498 ± 5.706; experimental treatment log CFU / g fruit = 2.963 ± 2.268). Decay rate at end of the first 24 h for the positive control is 0.910 and for the experimental treatment (*E*. *coli* applied via deer scat) is 0.133.

All the collected blueberries in the experimental scat treatment group and also in the positive control group were found to have non-pathogenic *E*. *coli* O157:H7. As expected, no *E*. *coli* was observed on the negative control plates during all time-periods (detection limit < 1.0 log CFU/g). When analyzed using a repeated measures MANOVA (independent factors: time and time*treatment), both the time (*F*_(1.9,18.8)_ = 678.884, *P* < 0.0001 (Greenhouse-Geisser Ɛ = 0.627) and the interaction term was significant (*F*_(1.9,18.8)_ = 358.486, *P* < 0.0001 (Greenhouse-Geisser Ɛ = 0.627). This analysis indicated that the treatments differed in mean log CFU *E*. *coli* levels and that these bacterial population differences were influenced by sampling time. Berry contamination in the positive control treatment was recorded at a concentration of log 6.50 CFU 2 h after application, while berries contacted by the inoculated scat were recorded at a contamination concentration of log 2.90 CFUs 2 h after the intial *E*. *coli* application. The difference in the initial *E*. *coli* levels in the to treatments was significantly different (Tukey HSD LS means test, *P* ≤ 0.05). The positive control treatment resulted in berries characterized by a very high decay rate (90% cumulative reduction in *E*. *coli* on berries) in the first 24 h after application to the blueberry plants, followed by a slower decay when bacterial densities reached a very low level from 24 to 72 h after application (90% to ca. 100% reduction in *E*. *coli* levels, Fig. 1). The scat inoculation treatment resulted in berries that showed an increasing decay of *E*. *coli* levels through the 72 h experiment, up to ca. 60% reduction in initial bacterial levels (Fig. 1). For both treatment groups (positive control and experimental scat inoculation), as time from inoculation increased, persistence of the bacteria decreased but at different rates (Fig. 1). At the end of the experiment, 72 h after *E*. *coli* application, despite the decay of *E*. *coli* in both treatments, there was a significant difference (Tukey HSD LS means test, *P* ≤ 0.05) in the *E*. *coli* log CFU/g concentration levels on berries (positive control = 3.183 ± 0.135 log CFU/g, experimental scat treatment = 2.368 ± 0.112 log CFU/g).

### Role of the dung beetle in pathogen transmission/suppression

#### Scat removal (lab experiment 1)

Beetles buried and degraded identical amounts of scat independent of scat inoculation with *E*. *coli* O157:H7 (29.2% and 29.2% respectively, n = 8, *F*_(1,6)_ < 0.001, *P* = 0.99). These data strongly suggest that the presence of *E*. *coli* O157:H7 did not affect the amount of attraction and utilization of deer scat by dung beetles.

#### Blueberry contamination (lab experiment 1)

No *E*. *coli* O157:H7 was detected on blueberries from any of the treatment groups. The likelihood of detecting dung beetles vectoring *E*. *coli* was maximized, as the 7 log CFU/ml inoculation level is slightly higher than levels of *E*. *coli* O157:H7 likely to be detected in nature [31]. If the dung beetle species, *Onthophagus hecate*, was prone to vector the pathogen to berries, these methods would have detected the pathogen on berry samples. Throughout the experiment, dung beetles were never observed in the blueberry canopy or on fruit, indicating that the generalist dung beetle, *Onthophagus hecate*, does not play a general role in vectoring the *E*. *coli* O157:H7 from inoculated white-tailed deer feces to ripe, pre-harvested, lowbush blueberries.

#### Soil contamination (lab experiment 1)

**Fig 2:**
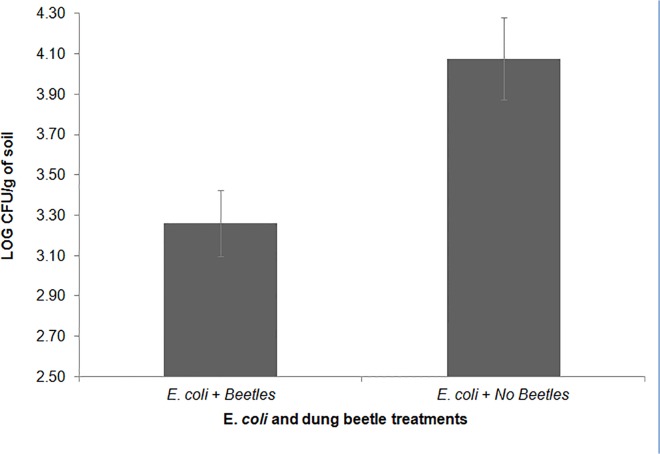
Laboratory experiment 1- Dung beetles drive number of E. coli colonies. Bacteria (log CFU/g, population values rank transformed) from soil samples subjected to different treatment combinations of beetles, and deer scat. Soil from microcosms including *E*. *coli* + dung beetles had significantly fewer bacterial colonies of than soil from microcosms containing *E*. *coli* + NO beetles (*F*_(2,9)_ = 7.757; *P* = 0.05). (Shown with means and SE bars).

Plates from the treatment including “*E*. *coli* inoculated scat and NO beetles” had a greater amount of colorless colonies than plates from the “*E*. *coli* inoculated scat WITH beetles” treatment (*F*_*(2*,*9)*_ = 7.757; *P* = 0.05, Fig. 2). As both treatments had *E*. *coli* inoculated scat, these data suggest that beetles play a role in decreasing the amount of bacteria including *E*. *coli* persisting in the soil (measured as bacterial CFUs). These data indicate that beetles have the ability to reduce the amount of bacteria in *E*. *coli* O157:H7-contaminated soils to levels not different from soils with no *E*. *coli* contamination.

#### Scat removal (lab experiment 2)

As anticipated, a significant difference in the amount of scat buried was dependent upon beetle presence (*F*_(2,17)_ = 4.102, *P* = 0.0379). In microcosms that include scat artificially inoculated with pathogenic *E*. *coli*, more burial of scat occurs when beetles are present than when beetles are not present (*P* = 0.0119). However, no significant difference in the amount of scat burial occurred between the treatment with *E*. *coli* inoculated feces and treatment with no added bacteria (*P* = 0.1477), indicating that beetles do not prefer to consume non-contaminated scat over contaminated scat, corroborating our findings previously shown in “*Scat removal results (lab experiment 1)”*.

#### Soil contamination (lab experiment 2)

**Fig 3:**
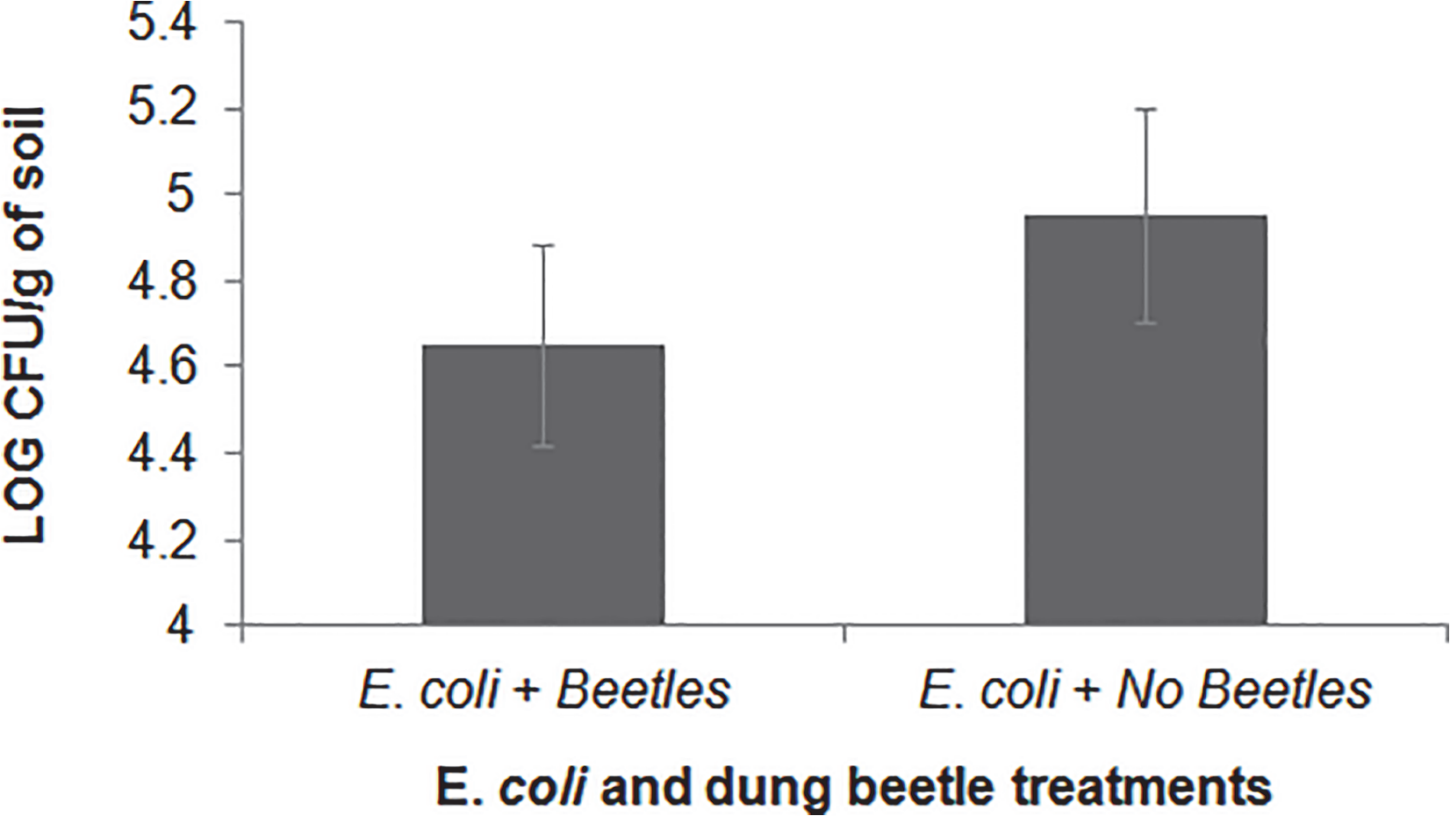
Laboratory experiment 2- Dung beetles affect number of *E*. *coli* colonies. Levels (log CFUs) of colorless bacterial colonies present within each treatment from lab experiment 2. Significant differences exist ((F_(2,17)_ = 8.062, *P* = 0.004)) indicating dung beetles can decrease the amount of *E*. *coli* present within the system (shown with means and SE bars).

When levels of *E*. *coli* were compared between treatments (data log transformed), a statistically significant difference was detected (*F*_(2,15)_ = 8.062, *P* = 0.004, Fig. 3). Significantly lower counts (log CFU) of *E*. *coli* were found within the microcosms with dung beetles present, as compared to those without dung beetles.

#### Beetle contamination (lab experiment 2)

No significant differences in the levels of bacteria (CFUs of colorless colonies) were found between the bodies of those dung beetles feeding on scat inoculated with *E*. *coli* O157:H7 and those beetles feeding on non-inoculated scat (*F*_(1,11)_ = 1.922, *P* = 0.196).

#### E. coli O157:H7 agglutination confirmation test (lab experiment 1 and 2)

Treatments including “*E*. *coli* O157:H7 inoculated scat and NO beetles” and “*E*. *coli* O157:H7 scat WITH beetles” both gave positive results in the agglutination assays. The control treatment “No *E*. *coli* O157:H7 scat with beetles” gave negative results in the agglutination assay, verifying that there was no inadvertent contamination in the experiments.

## Discussion

Results from the wildlife feces survey confirm that *E*. *coli* O157:H7 is present in wildlife scat within the lowbush blueberry agroecosystem (n = 318 samples, 1.88%). Interestingly, five of the six samples testing positive for *E*. *coli* O157:H7 were collected close to harvest time (early September), which could pose a potential food safety risk. However, with only 1.9% of all samples testing positive for the pathogen, it is unclear what the actual risk of food contamination is; it would be necessary to process thousands of scat samples and conduct sampling over several years in order to make a conclusive statement about the seasonality of the pathogen in the environment. Rasmussen and Casey [32] state that the prevalence of *E*. *coli* O157:H7 increases during the summer and early fall; however, this study focused on cattle and the seasonality of the pathogen is unknown with regards to white-tailed deer [11].

Despite the low prevalence of scat samples testing positive in the lowbush blueberry agroecosystem, our direct transmission field study confirms the plausibility of food contamination as a result of infected scat directly contacting berries following our simulation of defecation by deer. Previous studies regarding pathogen transmission to produce have dealt with either manure fertilizer applications [33–35] or investigatory research examining potential contaminants after an outbreak has occurred [7,8,36–38]. Though the risk of infection in the wildlife population appears to be low, the potential for food contamination remains. In the field study, rates of the attenuated pathogen decay in the environment were seen for both the positive control (sprayed directly on the berries) and experimental group (inoculated scat) indicating that, when exposed to environmental conditions; the level of *E*. *coli* will decline over time. However, different decay rates between the positive control and the experimental group were seen. The amounts of colonies detected were initially higher in the positive control group, but decreased more quickly than those occurring in the experimental group. In fact, nearly all of the *E*. *coli* populations in the positive control had been reduced within the first 24 h, while a large portion of the experimental group bacteria still remained. After 72 h, 40% of the experimental group *E*. *coli* persisted on the fruit. This finding raises the question of whether the increased decay rate of the positive control group is associated with any sort of density-dependent phenomenon. Though a rain event (79 mm) occurred between the 48 and 72 hr sampling periods, no marked shift in decay rates was directly apparent. Decay rates witnessed in this experiment are higher than the rate of *E*. *coli* O157:H7 reduction previously observed in ovine feces [26].

In a recent field study regarding dung beetle-mediated deer scat removal from blueberry fields [18], an average of 58.46% removal was observed (n = 164). Thus, the 30% removal of scat observed during the lab study is lower than that observed *in situ*. The number of beetles per available amount of scat may have also played a role in the amount consumed. The low number of dung beetles added to each microcosm may have limited feeding on scat. The laboratory experiment we designed precluded any scarab recruitment. In addition, while setting up the experiment, slight disturbance of the soil structure may have impacted the behavior of the beetles, causing them to feed at low rates. No studies to date describe how many individuals of this species of beetles would recruit to a given amount of deer scat or the functional response of these beetles to specific quantities of deer scat. Therefore, it is challenging to compare the removal rates observed in the lab with those realized in the field. Our study does, however, provide relative removal rates with which to better understand the feeding preference of these beetles.

While other studies have found dung beetles to offer a wide range of ecosystem services related to dung removal and pathogen suppression [13,15,16,39], our studies presented here demonstrate beneficial ecosystem services offered by dung beetles in the light of developing food safety concerns. The dung beetles in our study did not display a feeding preference between pathogen-laden feces and feces that had not been inoculated with *E*. *coli* O157:H7. The lower levels of *E*. *coli* O157:H7 recovered from microcosms containing beetles (compared to those to those lacking beetles) indicate that dung beetles are contributing to this decrease. We suspect that the physical manipulation of the scat, including burial, decreases pathogen persistence [26]. However, an alternative mechanism might be antimicrobial properties present on the carapace of the beetle, as found in ants [40]. Hwang et al. [41] isolated antimicrobial response genes responding to *E*. *coli* from the dung beetle *Copris triparititus* (Coleoptera: Scarabaeidae), indicating natural anti-microbial properties are possessed by at least some dung beetles. Due to the levels of exposure to many types of entomopathogenic bacteria, this is not an uncommon trait found in insects [42].

While detecting lower levels of the pathogenic *E*. *coli* in the presence of dung beetles is a novel and interesting finding, the fact that the beetles manipulated and fed on the scat without transmitting the pathogen to the fruit, is equally as important. Resulting lower levels of pathogenic *E*. *coli* in the soil due to dung beetle activity would not be such a useful ecosystem service if beetles were acting as a vector of the pathogen from scat to the fruit. While many others have addressed the reduction and fate of *E*. *coli* contamination in agroecosystems [43–45], these results help better assess risk of food contamination due specifically to wildlife fecal contamination as well as provide sound evidence for potential dung beetle-mediated biological control of this risk.

Given the large geographical distribution of *Onthophagus hecate* [21], results from this study should be broadly applicable to agroecosystems. It is not known how other dung beetle species would perform in comparison to this test species. Other dung beetle species collected in association with lowbush blueberry agriculture are: *Onthophagus nuchicornis*, *O*. *orpheus*, *Dialytes striatus*, *Acrossus rubipennis*, *Geotrupes splendidulus*, and *Odonteus liebecki* [18]. The behavior of different species of dung beetles can vary with respect to how the beetles feed on the feces (rollers, tunnelers, dwellers)[46]. Moreover, it is unknown how far they forage, how readily they fly in search of dung, and how prone they are to move to the vegetation after contacting contaminated dung.

Further research is needed in areas related to the interaction of dung beetles and feces-born pathogen suppression. Specific questions of interest are: What are the biological mechanisms for the suppression of the pathogen? What happens to pathogens once dung is buried? Can dung beetle feeding decrease the concentration of pathogens that leach from the soil and contaminate waterways? How does the differential feeding ecology/behavior of various guilds of dung beetles influence the degradation of wildlife and human pathogens?

## Supporting Information

S1 Dataset This contains Tables S1, S2 and S3. (PDF)
