# Supplementary material for: Suppression of Escherichia coli O157:H7 by Dung Beetles (Coleoptera: Scarabaeidae) Using the Lowbush Blueberry Agroecosystem as a Model System
Source: PLoS One. 2015 Apr 7;10(4):e0120904. doi: 10.1371/journal.pone.0120904 (PMC4388438; doi:10.1371/journal.pone.0120904)
Supplement: S1 Dataset — (PDF) [file pone.0120904.s001.pdf]

Table S1. Field Experiment- Reduction in *E. coli* levels over time.

**Treatment group**	**Average proportion reductions of *E. Coli* O157:H7 left on treatment groups in field inoculation study at different time periods**			
	2hrs	24hrs	48hrs	72hrs
Positive control	0	0.902	0.989	0.999
Scat inoculation	0	0.281	0.450	0.671

Table S2. Table of raw data for laboratory experiment 1-Dung beetles drive number of *E. coli* colonies

Treatment type	Levels of *E. Coli* O157:H7 (log CFU/g) present within each soil treatment from lab experiment 1
	**Average**
**Beetles + *E. coli* O157:H7**	3.26
***E. coli* O157:H7 + No Beetles**	4.08

Table S3.Table of raw data for laboratory experiment 2- Dung beetles affect number of *E. coli* colonies.

Treatment type	Levels of *E. Coli* O157:H7 (log CFU/g) present within each soil treatment from lab experiment 2<br>Average
**Beetles + *E. coli* O157:H7**	4.65
***E. coli* O157:H7 + No Beetles**	4.95
